# Safety assessment of symptomatic intracranial atherosclerotic stenosis: a comparison between sole balloon angioplasty and medical treatment

**DOI:** 10.3389/fneur.2025.1513086

**Published:** 2025-06-18

**Authors:** Wen-feng Cao, An Wen, Xian-min Cao, Ling-feng Wu, Yong-liang Zhou, Zheng-bing Xiang, Wei Rao, Quan-hong Chu, Wang-Wang Hong, Shi-min Liu

**Affiliations:** ^1^Department of Neurology, Jiangxi Provincial People's Hospital (The First Affiliated Hospital of Nanchang Medical College), Nanchang, Jiangxi, China; ^2^Department of Neurology, Xiangya Hospital, Central South University, Jiangxi (National Regional Center for Neurological Diseases), Nanchang, Jiangxi, China; ^3^Medical College of Nanchang University, Nanchang, Jiangxi, China

**Keywords:** symptomatic intracranial atherosclerotic stenosis, medical management, balloon angioplasty, stent, perioperative

## Abstract

**Background:**

Patients with symptomatic intracranial arterial stenosis (sICAS) are at risk of perioperative complications associated with stent placement and medication recurrence. Simple balloon angioplasty (SBA), a less invasive and safer alternative to stent placement, is an effective alternative treatment for sICAS.

**Methods:**

We conducted a retrospective analysis on patients with sICAS treated at the Jiangxi Provincial People’s Hospital between January 2020 and December 2023. Patients with severe stenosis (70–99%) were divided into the medical management (MM) and SBA groups. Demographics, medical histories, National Institutes of Health Stroke Scale (NIHSS) scores, vessel stenosis, postoperative residual stenosis, and 30-day outcomes were also assessed.

**Results:**

This study enrolled 176 patients, including 95 (66 males, mean age 57.4 ± 1.07 years) and 81 (55 males, mean age 61.1 ± 0.94 years) in the MM and SBA groups, respectively. Patients in the SBA group were significantly older than those in the MM group (*p* < 0.05). No significant differences were observed in sex, comorbidities (hypertension, diabetes, hyperlipidemia, smoking/alcohol use, and prior stroke), or baseline NIHSS scores (all *p* > 0.05). Pre-treatment stenosis rates were similar between groups: 80.90 ± 0.85% vs. 79.60 ± 1.01% (*p* > 0.05). One patient in the SBA group failed due to vessel tortuosity, while the remaining 80 procedures were successful. Of these, 15 patients (18.5%) required rescue stenting—11 because of elastic recoil and 4 because of flow-limiting dissection. The immediate residual stenosis rate was 24.68 ± 1.41%. Within 30 days, endpoint events occurred in four patients (4.2%) in the medical group (progressive infarction) and seven patients (8.6%) in the angioplasty group, including intracerebral and subarachnoid hemorrhage (*n* = 2), perforator infarction (*n* = 3), infarct progression (*n* = 1), and cortical infarction (*n* = 1). No deaths occurred in either group. The difference in the event rates was not statistically significant (*p* > 0.05). Subgroup analysis revealed that arterial dissection was significantly associated with postoperative endpoint events (*p* < 0.05), while occurrence was correlated with lesion length (*p* < 0.05), but not with the selected balloon size (*p* > 0.05). There was no significant difference in endpoint events between submaximal (< 90% of normal vessel diameter) and aggressive (> 90% of normal vessel diameter) angioplasties (*p* > 0.05).

**Conclusion:**

Overall, this study suggests that SBA does not significantly increase the 30-day risk of stroke or death in patients with sICAS compared with medical therapy. Both submaximal and aggressive angioplasty are safe. Further research is warranted to refine patient selection, optimize balloon size, and develop strategies to minimize the need for rescue stenting and reduce the risk of arterial dissection.

## 1 Introduction

Intracranial atherosclerotic stenosis (ICAS) is a major cause of ischemic stroke, particularly in Asian populations, where it accounts for a significant proportion of stroke-related morbidity (1). The management of symptomatic ICAS (sICAS) has been extensively studied. For example, the WASID study found that stroke or death rates within a year were 17% (warfarin) and 15% (aspirin), with a 22% recurrence rate over 1.8 years (2). Further, the Stenting vs. Aggressive Medical Management for Preventing Recurrent Stroke in Intracranial Stenosis (SAMMPRIS) and Vitesse Intracranial Stent Study for Ischemic Stroke Therapy (VISSIT) trials demonstrated that aggressive medical management (AMM) was superior to intracranial stenting due to high periprocedural risks (3, 4). More recently, the China Angioplasty and Stenting for Symptomatic Intracranial Severe Stenosis (CASSISS) trial re-evaluated the role of intracranial stenting by refining the patient selection criteria and utilizing more experienced surgeons. However, the CASSISS found no significant benefit of stenting plus medical therapy compared with medical therapy alone in reducing the risk of stroke or death (5).

Recently, simple balloon angioplasty (SBA) has gained clinical attention as a less invasive treatment option to reduce intravascular mechanical stimulation. Observational studies and meta-analyses have further shown that SBA may have a lower periprocedural complication rate in the treatment of sICAS, and has demonstrated favorable stroke prevention outcomes in several studies. This strategy offers superior short-term safety, and has considerable potential for long-term stroke prevention (6, 7). The 2024 Balloon Angioplasty versus Medical Management for Intracranial Artery Stenosis (BASIS) trial represents a significant advancement in this field (8). This study investigated whether the combination of balloon angioplasty and AMM is superior to AMM alone in the treatment of sICAS. BASIS also found that balloon angioplasty significantly lowered the risk of stroke or death within 12 months compared with AMM alone (4.4% vs. 13.5%, HR: 0.32, *p* < 0.001), indicating that balloon angioplasty is a safer and more effective option than medication or stenting, positioning it as a promising preferred treatment.

Although the BASIS trial offered high-quality evidence supporting the use of balloon angioplasty for the treatment of sICAS, its applicability in real-world clinical practice remains unclear. It is unclear whether the findings derived from the highly selected population in this randomized controlled trial could be generalized to broader patient populations and diverse clinical settings. In the BASIS trial, SBA was performed using balloons sized at 50–70% of the target vessel diameter; however, the safety and efficacy of these larger balloon sizes, which are more commonly used in real-world practice, have not yet been thoroughly evaluated. Therefore, the present study aimed to assess the periprocedural safety of balloon angioplasty compared with medical therapy in patients with sICAS in a non-randomized, real-world setting. Specifically, we explored whether balloons sized up to approximately 90% of the target vessel diameter could achieve comparable short-term safety outcomes. These findings provide evidence-based insights for future multicenter studies, and support the development of individualized treatment strategies.

## 2 Methods

This retrospective analysis was conducted on clinical data from patients who had experienced transient ischemic attacks or non-disabling ischemic strokes [modified Rankin scale score (mRS) ≤ 2] within 60 days at the Department of Neurology, Jiangxi Provincial People’s Hospital, from January 2020 to December 2023. All patients underwent cerebral angiography, confirming severe stenosis (stenosis rate, 70–99%). Based on whether they underwent balloon angioplasty, eligible patients were divided into the medical management (MM) and SBA groups. Preoperative ischemic symptoms in the SBA group remained stable for at least 7 days. Prior to obtaining informed consent, the patients or their families were informed about the surgical risks.

The inclusion criteria were as follows: 1. Age 18–85 years; 2. had primary or recurrent sICAS [a recent TIA (<90 days) or ischemic stroke (14–90 days)] before enrollment; 3. Cerebral angiography confirmed major intracranial vessel involvement (intracranial segments of the internal carotid artery, middle cerebral artery, intracranial segments of the vertebral artery, and basilar artery), measured using the WASID method (9), with stenosis severity ranging from 70 to 99% and vessel diameter ≥1.5 mm; 4. Preoperative mRS score ≤2 points. Conversely, the exclusion criteria were: 1. History of intracranial bleeding; 2. Uncontrolled hypertension before treatment, blood pressure >185/110 mmHg; 3. Active bleeding or bleeding tendency; 4. Severe heart, liver, or kidney dysfunction; 5. Non-atherosclerotic causes of stenosis; 6. Hemoglobin <100 g/L, platelet count <100 × 10^9^/L, coagulation dysfunction, or uncontrollable bleeding factors; 7. Intolerance to antiplatelet drugs; 8. Incomplete clinical data or inability to cooperate with follow-up.

Patients in the MM group received dual antiplatelet therapy comprising aspirin (100 mg/day), clopidogrel (75 mg/day), or ticagrelor (90 mg/day, twice daily). Upon admission, all patients underwent CYP2C19 gene detection by polymerase chain reaction (PCR) with a fluorescent probe. If a patient did not carry the CYP2C19 loss-of-function allele, the treatment of choice comprised a combination of aspirin and clopidogrel. If a patient carried the CYP2C19 loss-of-function allele, the treatment of choice comprised a combination of aspirin and ticagrelor (10).

In the SBA group, patients received a minimum of 3 days of dual antiplatelet therapy prior to the procedure. Anesthesia (local or general) was administered by a surgeon. The Seldinger technique was used for femoral artery puncture and an 8F guiding catheter was employed, with the option of a 6F long sheath or an 8F guiding catheter plus an intermediate catheter. The microcatheter, guided by a 0.014-inch 2 m micro-guidewire, traversed the lesion to reach the distal end. After replacing the initial guidewire with a 3 m micro-guidewire (for precise navigation), the microcatheter was withdrawn. The chosen balloon (SacSpeed, Acandis, China; Gateway, Stryker, USA; Neuro RX, SinoMed, China) was subsequently advanced along the micro-guidewire to the lesion site. Balloon selection was as follows: If the diameter of the target vessel was between 1.5–2.5 mm, the balloon size was selected to be 80–125% of the vessel diameter; if the target vessel diameter was > 2.6 mm, the balloon size was selected to be 60–100% of the vessel diameter. The balloon was slowly inflated once (30–60 s/atmospheric pressure), reaching nominal pressure, followed by a 1–3 min dwell time and slow deflation (5–10 s/atmospheric pressure). If the remaining stenosis was more than 50%, a second or third dilation was considered, or a larger balloon was chosen. The physician subsequently performed a follow-up angiogram 15–20 min later and the procedure was considered successful if the residual stenosis was less than 50%, the procedure is deemed successful. As a rescue strategy, stenting (APOLLP, MicroPort, China; Neuroform EZ, Stryker, USA; Enterprise, Johnson & Johnson, USA) may be used at the operator’s discretion under the following conditions: 1. elastic recoil ≥50%; 2. the occurrence of dissection at the lesion site with extended thrombolysis in cerebral infarction (eTICI) score <3 (11).

The clinical data collected from both groups included sex, age, medical history (hypertension, diabetes, hyperlipidemia, smoking and drinking, and history of stroke), National Institutes of Health Stroke Scale (NIHSS) score at onset (total score ranging from 0 to 42) (12), responsible vessels, degree of vascular stenosis, and treatment modality (medical treatment, balloon angioplasty, and stent rescue treatment). Additionally, information on anesthesia methods, balloon size and length, posttreatment outcomes (including immediate residual stenosis rate), and postoperative follow-up status was obtained. Patients were followed-up on postoperative days 1, 7, 15, and 30 via outpatient visits, telephone communication, and readmission, as necessary.

The observation period involved vigilant monitoring for any stroke-related events occurring within 30 days after the procedure. This included ischemic stroke, intracerebral hemorrhage, subarachnoid hemorrhage, and progressive stroke (defined as an increase of ≥2 points in the NIHSS score during the follow-up period) (13, 14). Mortality outcomes were documented and tracked.

This study was approved by the local Research Ethics Committee (No: 2022–042) and adhered to the principles of the Declaration of Helsinki. Informed consent for the publication of data was obtained from the patients or their family members.

### 2.1 Statistical analysis

Data analysis was conducted using SPSS 22.0 statistical software. Normal distribution of continuous data was visually assessed using graphs and histograms. Normally distributed continuous data are reported as the mean ± standard deviation (x ± s), while non-normally distributed continuous data are presented as the median and quartiles [*M*(*P*_25_, *P*_75_)]. Group comparisons were performed using independent sample *t*-tests for normally distributed data, or Mann–Whitney U tests for non-normally distributed data. Categorical data are expressed as frequencies and percentages (%). Group comparisons for categorical data were conducted using the χ^2^-test, continuous correction χ^2^-test, or Fisher’s exact test, with statistical significance set at *p* < 0.05.

## 3 Results

Overall, 176 patients were included in the study, including 121 males and 55 females, with an age range of 37 to 81 years and a mean age of (59.07 ± 0.73) years. The National Institutes of Health Stroke Scale (NIHSS) score at stroke onset was 1 (0, 3). Medical history revealed 59 cases of smoking (33.5%), 36 of alcohol consumption (20.5%), 127 had hypertension (72.2%), 55 had diabetes (31.3%), 85 had hyperlipidemia (48.3%), and 38 of previous stroke (21.6%).

As shown in Table 1, the MM group included 95 cases, consisting of 66 males and 29 females, with ages ranging from 37 to 81 years and a mean age of (57.4 ± 1.07) years. The SBA group comprised 81 cases, with 55 males and 26 females, aged between 45 to 80 years, and with a mean age of (61.1 ± 0.94) years.

**Table 1:** Baseline characteristics comparison between the medical management and simple balloon angioplasty group.

	Medical management group (*n* = 95)	Simple balloon angioplasty group (*n* = 81)	Statistics	*P*
Age (y, x ± s)	57.4 ± 1.07	61.1 ± 0.94	2.551	0.012
Male, n (%)	66 (69.5)	55 (67.9)	0.05	0.823
Hypertension, n (%)	70 (73.7)	57 (70.4)	0.239	0.625
Diabetes mellitus, n (%)	27(28.4)	28(34.6)	0.769	0.381
Hyperlipidemia, n (%)	42(44.2)	43(53.1)	1.397	0.240
Prior stroke history, n (%)	17 (17.9)	21 (25.9)	0.825	0.364
Smoking, n (%)	29(30.5)	30(37.0)	0.832	0.362
Alcohol consumption, n (%)	18(18.9)	18(22.2)	0.288	0.591
NIHSS at stroke onset M (Q25, Q75)	2 (0, 3)	1 (0, 3)	−0.405	0.686
Responsible vessels, n (%)			1.901	0.593
Intracranial of ICA	11 (11.6)	9 (11.1)		
MCA	55 (57.9)	50 (61.7)		
BA	22 (23.1)	13 (16.1)		
V4 of the VA	7(7.4)	9(11.1)		
Stenosis degree, %	80.9 ± 0.85	79.6 ± 1.01	0.965	0.336

NIHSS, national institute of health stroke scores; ICA, internal carotid artery; MCA, middle cerebral artery; BA, basilar artery; VA, vertebral artery.

**Figure 1:**
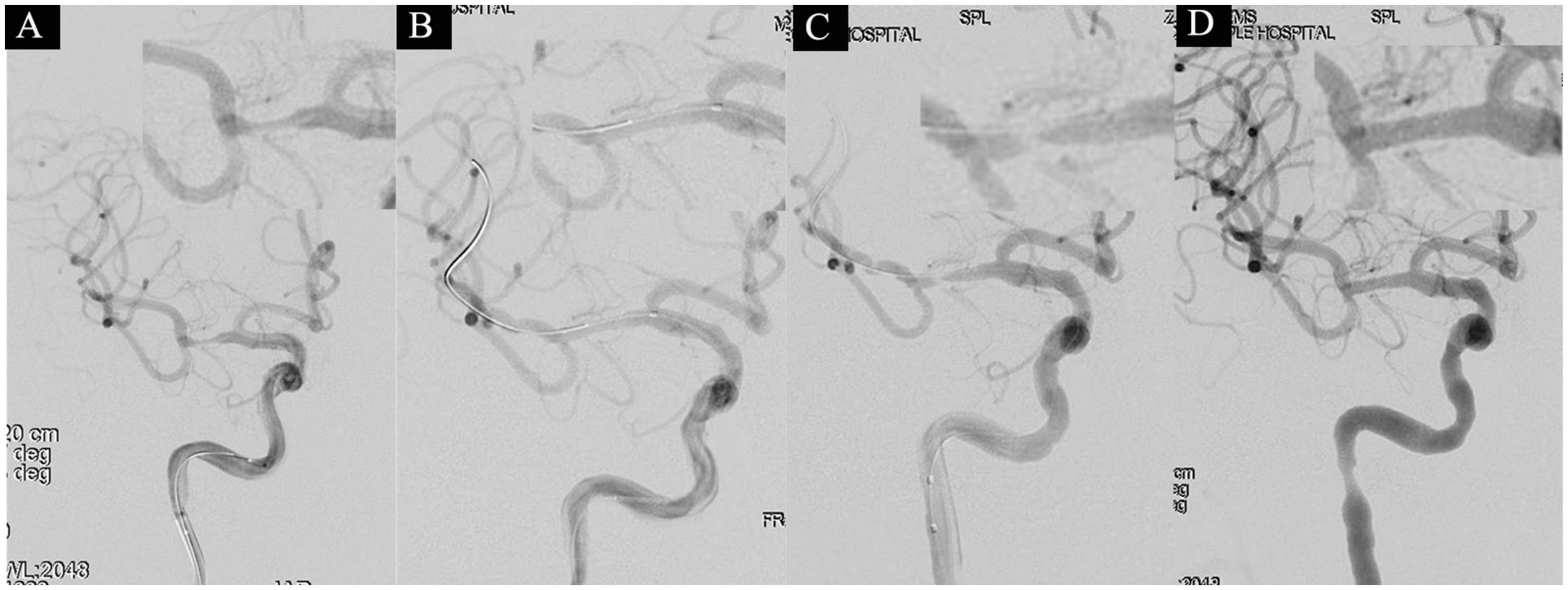
A 53-year-old male with a 3-year history of diabetes was hospitalized on July 3, 2021, presenting with a 2-day history of left-sided limb weakness and numbness. His NIHSS score at admission was 2 points (facial palsy 1 point, dysarthria 1 point). Diagnostic head MRI identified an acute infarction in the right cerebral hemisphere. On July 19 (after 18 days of stroke onset), the patient underwent a balloon angioplasty under general anesthesia to address severe stenosis in the M1 segment of the right MCA. The proximal lumen of the stenosis measured 2.5 mm, with the narrowest part at 0.5 mm, indicating an 80% stenosis degree **(A)**. Following consecutive use of 2.0 × 15 mm and 2.5 × 10 mm balloons, a subsequent angiogram exhibited noticeable improvement in the stenosis **(B)**. However, after a 10-min observation period, there was observed restenosis at the stenotic site **(C)**. Subsequently, a 2.5 × 10 mm balloon-mounted stent (APOLLP, MicroPort, China) was deployed, effectively relieving the stenosis and achieving an eTICI grade of 3 **(D)**.

**Figure 2:**
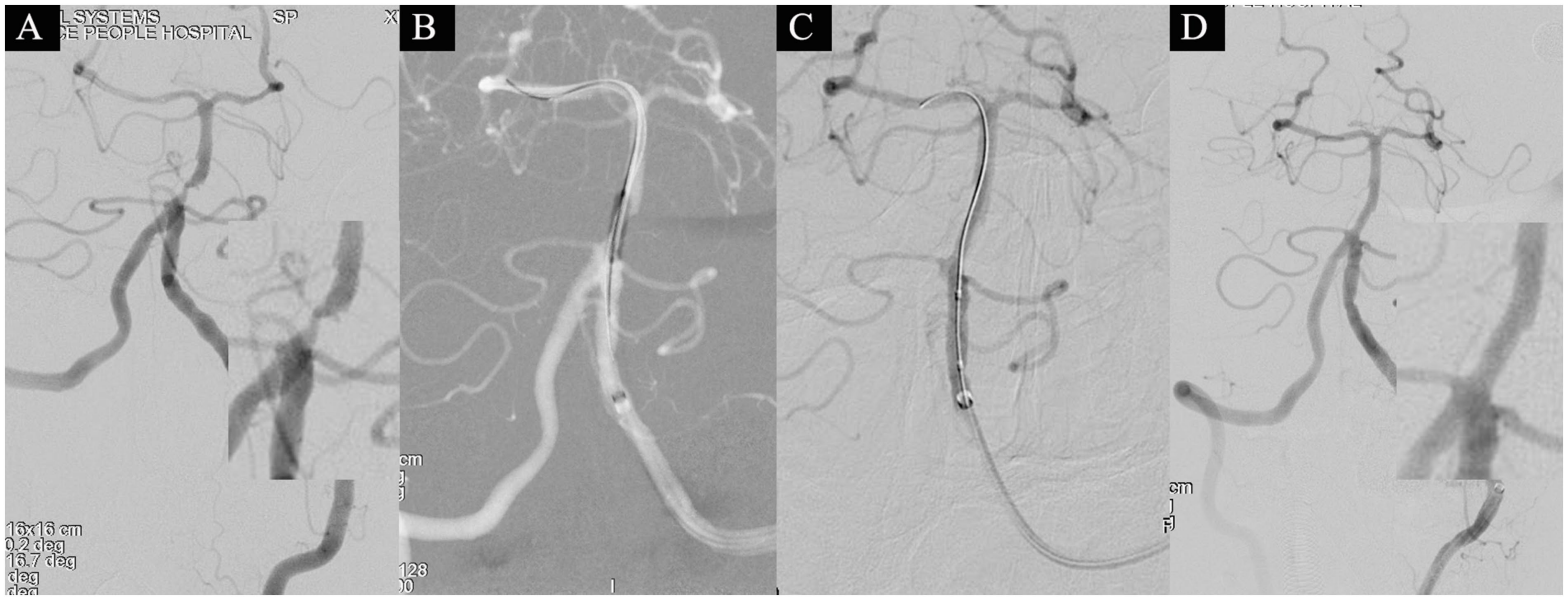
A 58-year-old male presented with sudden-onset right-sided weakness for 12 days and was admitted on November 4, 2021. Head MRI revealed a left pontine infarction, with an NIHSS score of 1 point at admission (attributed to dysarthria). On November 8th (after 16 days of stroke onset), the patient underwent a comprehensive basilar artery angioplasty procedure. Angiography identified severe stenosis in the basilar artery, characterized by a distal lumen of 2.9 mm, the narrowest segment measuring 0.4 mm, and an overall stenosis degree of 86.2% **(A)**. Subsequent dilations were conducted using 1.5 × 15 mm and 2.5 × 15 mm balloons **(B,C)**. Approximately 10% residual stenosis remained. Following a 15-min observation period, a subsequent angiogram exhibited no significant restenosis **(D)**, resulting in the attainment of eTICI grade 3.

**Figure 3:**
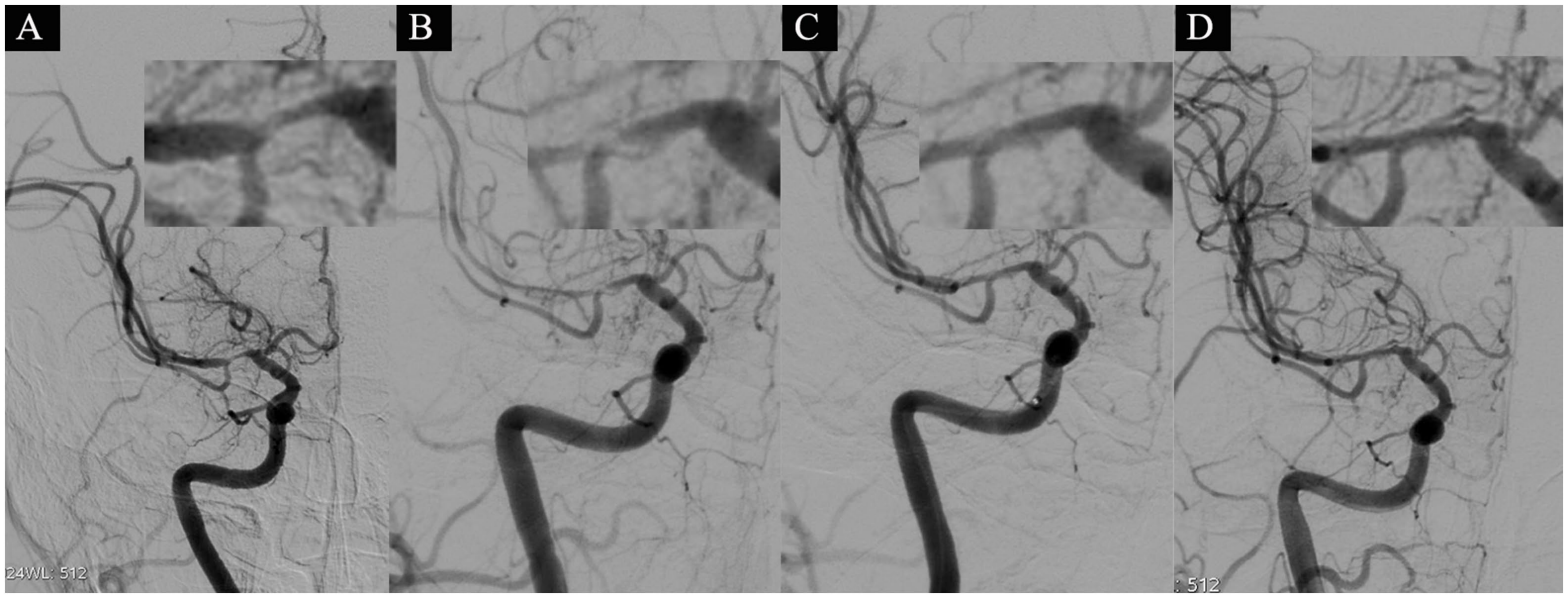
A 64-year-old female presented with acute-onset left-sided limb numbness, weakness, and slurred speech, prompting admission on March 7, 2020. The NIHSS score at admission was 5 (facial palsy 2, sensory deficits1, language impairment 1, dysarthria 1). Head MRI revealed an acute ischemic stroke adjacent to the right lateral ventricle. On March 25(after 19 days of stroke onset), cerebral angiography revealed severe stenosis with a distal diameter of 2.0 mm, a narrowest segment of 0.5 mm, and a stenosis degree of 75% **(A)**. Subsequent dilation with a 1.5 × 15 mm balloon led to an arterial dissection **(B)**. A slow infusion of 5 mL of tirofiban was administered through the artery, and after a 15-min observation, the dissection showed improvement, achieving eTICI grade 3 blood flow **(C,D)**.

**Figure 4:**
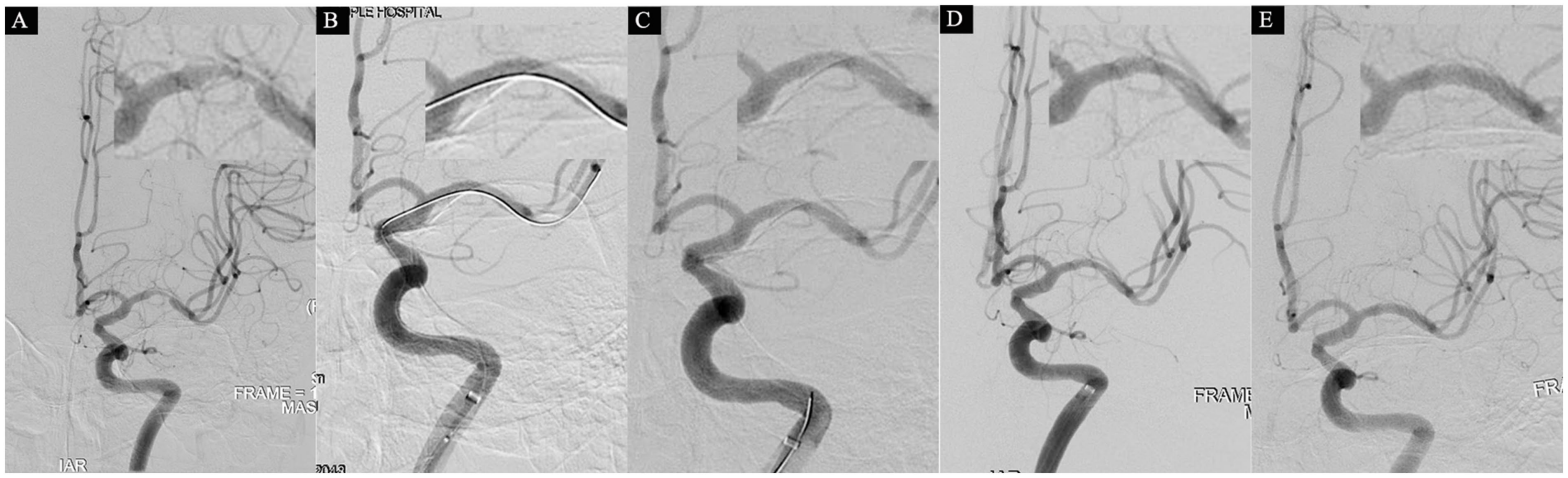
A 75-year-old male who was admitted on June 28, 2021, with a 22-day history of right-sided limb weakness. At admission, the NIHSS score was 0, and head MRI showed no evidence of acute infarction, leading to a diagnosis of transient ischemic attack (TIA). Subsequent cerebral angiography on June 30 revealed severe stenosis in the M1 segment of the right middle cerebral artery (MCA), characterized by a proximal lumen of 2.8 mm, the narrowest part at 0.6 mm, and a stenosis degree of 79% **(A)**. An initial attempt at dilation was made using a 2.0 × 15 mm balloon, resulting in satisfactory reconstruction of the stenosis **(B)**. However, upon withdrawal of the microguidewire for a follow-up angiogram, an unexpected dissection in the MCA was observed **(C)**. After a 10-min observation, a subsequent angiogram indicated a more significant dissection, along with sluggish blood flow in the distal MCA **(D)**, eTICI grade 2c. Consequently, a strategic decision was made to deploy a 3.0 × 13 mm balloon-mounted stent (APOLLP, MicroPort, China). A follow-up angiogram demonstrated successful eTICI grade 3 **(E)**.

The age of the patients in the SBA group was significantly higher than that in the MM group (*p* < 0.05); however, no statistically significant differences were observed between the two groups in terms of sex, medical history (hypertension, diabetes, hyperlipidemia, smoking, alcohol history, and stroke history), NIHSS score at onset, or degree of vascular stenosis (all *p* > 0.05). The predominant involvement of the responsible vessels in both groups was observed in the middle cerebral artery, with no statistically significant difference in the distribution of the responsible vessels between the two groups (*p* > 0.05) (Figures 1–4).

As shown in Table 2, In the SBA group, one patient did not complete balloon dilation due to severe vessel tortuosity, whereas the remaining patients successfully underwent the procedure, with 13 and 68 patients under local and general anesthesia, respectively. The time from the last onset to surgery was 23.35 ± 1.83 days, the target vessel diameter was 2.50 ± 0.06 mm, the selected balloon size was 2.26 ± 0.40 mm, and the balloon length was 13.64 ± 0.29 mm. Overall, 15 patients underwent stent rescue, including 11 cases of >50% elastic recoil stenosis after balloon dilation and 4 cases of dissection with compromised distal perfusion (eTICI <3). Additionally, dissection occurred in nine other patients post-dilation; however, as the distal blood flow was preserved (eTICI = 3), stent placement was not required. The postoperative residual stenosis rate for all surgical patients was (24.68 ± 1.41) %.

**Table 2:** Clinical and procedural parameters in the simple balloon angioplasty group.

Time from onset to intervention, days	23.35 ± 1.83
Anesthesia method	
Local anesthesia	16% (13/81)
General anesthesia	84% (68/81)
Target vessel diameter, mm	2.5 ± 0.06
Balloon angioplasty only	80% (65/81)
Balloon size, mm	2.26 ± 0.40
Balloon length, mm	13.64 ± 0.29
Stent rescue	18.5% (15/81)
Post-dilation elastic recoil	11
Arterial dissection	4
Stenosis degree after intervention, %	24.68 ± 1.41%

During the 30-day follow-up, four patients (4.2%) in the MM group exhibited symptomatic progression. In the SBA group, seven patients (8.6%) experienced endpoint events, with five patients developing new-onset infarction in the surgical vascular supply area and one case of subarachnoid hemorrhage. Statistical analysis revealed no significant difference in the occurrence of endpoint events between the two groups (χ^2^ = 0.078, *p* = 0.779). No deaths occurred in either group.

In the comparison between the stent rescue and non-stent rescue patients within the SBA group, it was found that the absolute vessel diameter in the stent rescue patients was larger than that in the non-stent rescue patients 2.7(2.4, 3.2) mm vs. 2.4(2.1, 2.9) mm (*p* = 0.058, Table 3). Additionally, the balloon size used during treatment was significantly larger in the stent rescue patients than in non-stent rescue patients 2.5 (2, 3) mm vs. 2 (12, 15) mm (*p* = 0.018; Table 3).

**Table 3:** Comparison between stent-rescue and non-rescue patients in the simple balloon angioplasty.

	Stent rescue (*n* = 15)	Non-stent rescue (*n* = 65)	Statistics	*P*
Endpoint events, n (%)	2 (13.3)	5(7.7)	–	0.61
Responsible vessel (posterior circulation), n (%)	7 (46.7)	15 (23.1)		0.105
Target vessel diameter, mm	2.7(2.4, 3.2)	2.4(2.1, 2.9)	−1.895	0.058
Degree of stenosis, mm	79.27 ± 2.06	79.82 ± 1.16	0.209	0.835
Length of stenosis, mm	5.5(4, 7)	5.5(4, 8)	−0.068	0.946
Balloon size, mm	2.5(2, 3)	2(2, 2.5)	−2.361	0.018
Balloon length, mm	15(15, 15)	15(12, 15)	−1.847	0.065

When comparing dissection and non-dissection patients following balloon angioplasty, the stroke event rate was significantly higher in the dissection patients than in the non-dissection patients (*p* = 0.012) (Table 4). Furthermore, the length of the stenosis in the responsible vessel was significantly greater in patients with dissection than in those without dissection [6(5.3, 9.5) mm vs. 5(4. 7) mm] (*p* = 0.022) (Table 4).

**Table 4:** Comparison between patients with and without dissection in the simple balloon angioplasty.

	Dissection (*n* = 13)	Non-dissection (*n =* 67)	Statistics	*P*
Endpoint events, n (%)	4(30.8)	3(4.5)	–	0.012
Responsible vessel (posterior circulation), n (%)	4 (30.8)	18 (26.9)		0.745
Target vessel diameter, mm	2.4(2.1, 3.5)	2.5(2.2, 2.8)	0.000	1.0
Degree of stenosis, mm	81.92 ± 2.34	79.28 ± 1.13	0.947	0.342
Length of stenosis, mm	6.5(5.3, 9.5)	5(4, 7)	−2.290	0.022
Balloon size, mm	2.0(2, 2.8)	2.25(2.25, 2.5)	−0.055	0.956
Balloon length, mm	15(12, 15)	15(12, 15)	−1.014	0.310

According to the ratio of selected balloon diameter to target vessel diameter, Submaximal angioplasty was defined as a balloon-to-vessel diameter ratio <90%, and aggressive angioplasty as ≥90%. The aggressive and submaximal groups included 42 and 38 patients, respectively. There were no statistically significant differences between the two groups in terms of endpoint events, degree of stenosis, balloon size and length, or residual stenosis and dissection after the procedure (*p* > 0.05) (Table 5). However, the submaximal group had a significantly higher proportion of posterior circulation involvement as well as greater target vessel diameter and lesion length than the aggressive group (*p* < 0.05) (Table 5).

**Table 5:** Comparison between aggressive and submaximal angioplasty in the simple balloon angioplasty.

	Aggressive angioplasty (*n* = 42)	Submaximal angioplasty (*n* = 38)	Statistics	*P*
Endpoint events, n (%)	3 (7.1)	4 (10.5)		0.703
Responsible vessel (posterior circulation), n (%)	6 (14.3)	16 (42.1)	1.744	0.005
Target vessel diameter, mm	2.2(2,2.5)	2.8(2.4,3.3)	−4.681	0.000
Degree of stenosis, mm	78.81 ± 1.47	80.71 ± 1.40	−0.933	0.354
Length of stenosis, mm	4.75(3.5, 7.1)	5.8(5, 8)	−2.030	0.042
Balloon size, mm	2.25(2, 2.5)	2.25(2, 2.5)	−0.449	0.654
Balloon length, mm	15(12, 15)	15(12, 15)	−1.154	0.249
Dissection, n (%)	5 (11.9)	8 (21.1)	1.277	0.268
Stent rescue, n (%)	8 (19.0)	7 (18.4)	0.005	0.943
Stenosis degree after intervention, %	22.95 ± 1.89	26.58 ± 2.08	−1.292	0.200

## 4 Discussion

Overall, this retrospective study analyzed 176 patients with sICAS, of whom 81 underwent submaximal balloon angioplasty, with 80 procedures deemed successful. This study provides real-world evidence regarding the safety of SBA compared with medical therapy. The results showed that SBA did not increase the risk of periprocedural cerebral infarction or intracranial hemorrhage, further supporting its safety as a treatment option for sICAS, which is in line with the findings of the BASIS trial. However, arterial dissection occurred in 13 patients (16.3%) in the SBA group, with 4 requiring rescue stenting. An additional 11 patients underwent stent placement because of significant elastic recoil, indicating that SBA alone may be insufficient for certain complex lesions.

In the present study, seven out of 81 patients (8.6%) in the SBA group experienced endpoint events, including one case of intracerebral hemorrhage, one of subarachnoid hemorrhage, three of perforator infarctions, one of infarct enlargement, and one of cortical infarction (attributed to thrombus migration). The incidence of endpoint events in this study appears to be higher than the 4.4% reported in the BASIS trial. This discrepancy may be partly attributed to the use of relatively larger balloons in our study, with the average balloon diameter reaching 90.4% of the target vessel diameter (2.26 mm/2.50 mm), exceeding the 50–70% ratio recommended in the BASIS trial (8). The larger balloon size may have contributed to the three cases of perforator infarction via the “snowplowing effect” (15). Standard vessel diameter assessments in intracranial stenosis rely on the WASID or North American Symptomatic Carotid Endarterectomy Trial (NASCET) methods (16), with some studies exploring optical coherence tomography (OCT) (17). However, these methods are subject to measurement errors, particularly in the presence of vascular remodeling (negative or positive) (18). In proximal lesions, negative remodeling of the distal reference segments may have lead to undersized balloon selection using the WASID criteria. Therefore, individualized and accurate balloon sizing is essential to optimize the safety and efficacy of submaximal angioplasty. Another contributing factor was the limited size of the balloon. Currently, most commercially available balloons are manufactured in size increments of 0.25 mm or 0.5 mm, which may restrict precise matching to vessel diameter (19).

In the present study, arterial dissection occurred in 16.3% (13/80) of patients undergoing balloon angioplasty. Subgroup analysis revealed that patients who underwent dissection had a significantly higher rate of endpoint events than those without dissection, indicating a potential negative impact on periprocedural outcomes. Dissection was not associated with the relative balloon-to-vessel diameter, but was significantly correlated with lesion length (6.5 mm vs. 5 mm), indicating that longer stenotic segments may predict a higher dissection risk. Prior studies have reported similar dissection rates: 13% in a recent meta-analysis (20), 9.3% in submaximal angioplasty studies (21), and 14.5% in the BASIS trial (8). However, the BASIS trial did not analyze the clinical outcomes of patients who underwent dissection. However, the long-term effects of this dissection remain unclear. In coronary artery disease, dissection following drug-coated balloon angioplasty has not been associated with poor outcomes, although it is more common in patients with longer lesions (22). These findings underscore the need for further studies to clarify the short- and long-term implications of dissection during submaximal angioplasty.

In the treatment of sICAS, SBA may be associated with a higher risk of restenosis owing to the lack of sustained radial support and the potential for elastic recoil. A long-term follow-up study from Japan involving 72 sICAS patients reported a restenosis rate of 31.9% (23/72) within 6 to 111 months post-procedure. Seyed et al. conducted a systematic review of 15 retrospective studies on SBA, finding an overall restenosis rate of 20% (95% CI, 12–30%) (15). Restenosis following stent placement is another concern. The VISSIT trial reported a 1-year restenosis (defined as >50% luminal narrowing) rate of 26.5% (9/34), following balloon-expandable stent implantation (4). The WOVEN trial reported a 17.6% rate (18/102) at 1 year after Wingspan stent placement using a threshold of >70% stenosis (23). In the BASIS trial, among patients in the SBA group who completed cerebrovascular imaging at 1-year follow-up, 15.7% (24/153) developed restenosis, defined as >70% stenosis or a ≥ 30% increase from baseline. However, 38.6% (96/249) of patients were lost to follow-up, potentially introducing bias (8). Compared with stenting, SBA offers several biomechanical advantages. Namely, it avoids the persistent radial force exerted by stents and helps to preserve the native endothelium, which may theoretically reduce neointimal hyperplasia and lower the risk of restenosis (24). Nevertheless, apart from the BASIS trial, which is a prospective randomized controlled trial (RCT), most existing data on SBA have been derived from retrospective studies. Therefore, the true restenosis rate after SBA remains uncertain and warrants further validation in high-quality RCTs.

## 5 Conclusion

In conclusion, this study indicates that balloon angioplasty does not significantly increase the 30-day risk of stroke or death in patients with sICAS compared with medical therapy. Both submaximal and aggressive angioplasty are safe. Intraoperative arterial dissection was associated with periprocedural ischemic events, and lesion length significantly correlated with the occurrence of dissection. Even so, future long-term follow-up studies are still needed to evaluate the long-term efficacy and safety. Meanwhile, with the deepening understanding of the pathophysiology of intracranial artery stenosis, we can provide precise preoperative imaging assessment for patients with sICAS by means of imaging assessment techniques such as Computational Fluid Dynamics (CFD) and high-resolution magnetic resonance. Furthermore, with the continuous evolution and development of neurointerventional materials, we can optimize the selection of balloons and formulate the best interventional strategy to reduce the risk of arterial dissection and minimize the need for stent rescue treatment. We believe that more evidence will continue to emerge in the future, adding more evidence to the endovascular treatment of sICAS.

## Data Availability

The original contributions presented in the study are included in the article/supplementary material, further inquiries can be directed to the corresponding author.
